# Synthesis of dendritic silver nanostructures supported by graphene nanosheets and its application for highly sensitive detection of diazepam

**DOI:** 10.1016/j.msec.2015.07.037

**Published:** 2015-12-01

**Authors:** Mir Reza Majidi, Seyran Ghaderi, Karim Asadpour-Zeynali, Hossein Dastangoo

**Affiliations:** Department of Analytical Chemistry, Faculty of Chemistry, University of Tabriz, Tabriz, Iran

**Keywords:** Ag nanodendrimers, Graphene nanosheets, Electroreduction, Diazepam

## Abstract

In this paper, preparation, characterization and application of a new sensor for fast and simple determination of trace amount of diazepam were described. This sensor is based on Ag nanodendrimers (AgNDs) supported by graphene nanosheets modified glassy carbon electrode (GNs/GCE). The AgNDs were directly electrodeposited on the surface of electrode via potentiostatic method without using any templates, surfactants, or stabilizers. The structure of the synthesized AgNDs/GNs was characterized by scanning electron microscopy (SEM), energy dispersive X-ray (EDX) analysis, X-ray diffraction (XRD) and electrochemical impedance spectroscopy (EIS) techniques. The nanodendrimers with tree-like and hierarchical structures have a fascinating structure for fabrication of effective electrocatalysts. The experimental results confirmed that AgNDs/GNs/GC electrode has good electrocatalytic activity toward the reduction of diazepam. A low detection limit of 8.56 × 10^− 8^ M and a wide linear detection range of 1.0 × 10^− 7^ to 1.0 × 10^− 6^ M and 1.0 × 10^− 6^ to 20 × 10^− 6^ M were achieved via differential pulse voltammetry (DPV). The proposed electrode displayed excellent repeatability and long-term stability and it was satisfactorily used for determination of diazepam in real samples (commercially tablet, injection and human blood plasma) with high recovery.

## Highlights

• Applying a simple, fast and cost-effective method for synthesis of silver nanodendrimers
• Characterization of AgNDs/GNs/GCE surface by SEM, EDX, XRD, EIS and CV methods
• Successful application of this sensor for diazepam determination with an excellent detection limit
• Calculation of diffusion coefficient, electron transfer coefficient and standard heterogeneous rate constant for diazepam
• Satisfactorily using of this sensor for diazepam determination in several real samples

## Graphical abstract

The typical images for bare GC electrode, GNs/GCE and AgNDs/GNs/GCE and electrocatalytic reduction of diazepam on the surface of modified electrode.

**Figure:**
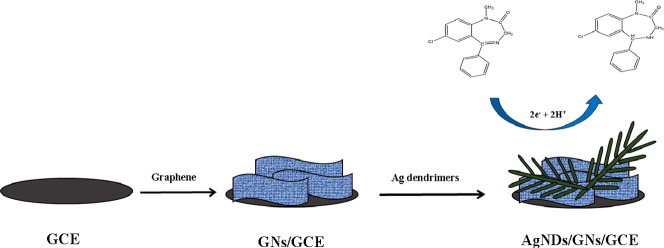

## 1 Introduction

Nowadays, nanotechnology is one of the most exciting fields in chemistry, physics, engineering and biology. Nanotechnology involves manufacturing of materials, structures, devices and systems through control of matter at the 1–100 nm scale [1], [2].

The nanoscale materials show unique and considerable characteristics, such as the catalytic activities, optical, electronic and magnetic properties that cannot be observed by their macro scaled counterparts [3]. Nanostructure materials have been found application as new efficient morphology of electrodes in electrochemical sensors and biosensors [4], [5]. The performance and stability of electrodes greatly have been changed along small changes to the surface of electrodes. When the dimensions of electrodes become smaller, the surface area-to-volume ratio of electrode enhances. Also, this leads to an increase in rate of electron transfer and improvement in mass transfer through enhanced diffusion [6].

In recent years, metals nanoparticles including Au, Pt, Pd, Cu, Ni and Ag have been extensively used as the electrode modifier for developing novel electrochemical sensors [7], [8], [9], [10].

On the other hand, morphology of metallic nanostructures has significant impacts on their electrocatalytic activity [11]. Various nanostructures such as nanotubes [12], [13] nanowires [14], [15], nanoflowers [16], [17] and nanorods [18], [19] have been introduced as different morphologies of the metal nanostructures in the electrochemical sensors.

Among a variety of morphologies, three-dimensional (3D) nanodendrimers with tree-like structures have a particular attractive structure for application in the catalysis and technological fields [20], [21], [22]. Because of unique properties such as hierarchical structures, high specific surface area, numerous active sites and sharp edges, nanodendrimers have been found promising applications in the electrochemical systems [23], [24].

For instance, Sawangphruk et al. fabricated a metallic silver nanodendrite modified reduced graphene oxide electrode with open pore structure by an electrodeposition method for H_2_O_2_ detection [21]. Chen and co-workers developed an enzyme-free nitrite sensor based on the electrodeposition of RGO and Cu nanodendrites on a glassy carbon electrode [25]. Wang et al. synthesized gold nanodendrites by one-step electrodeposition which was used to detect trace amounts of formaldehyde in indoor air [26].

Electrodeposition has arisen as a versatile and controllable technique to synthesis of metal nanostructures with high efficiency. This technique is simple, surfactant-free, cost-effective and time-saving one. It also allows size, shape and composition of particles to be adjusted by changing electrolyte composition, time duration and deposition potential [27], [28], [29].

Graphene nanosheet (GN) is a two-dimensional sheet of carbon atoms in a hexagonal configuration with atoms bonded by sp^2^ bonds [30]. Graphene exhibits unusual properties such as excellent electrical conductivity, large specific surface area, high electron mobility and high thermal conductivity. Because of these properties, graphene is an attractive candidate in analytical chemistry (e.g., for electrochemical catalysis and biosensing) [31], [32].

Diazepam (DZP) [7-chloro-1, 3-dihydro-1-methyl-5phenyl-2H-1, 4-benzodiazepin-2-one] is a medication that belongs to a class of drugs called benzodiazepines [33]. Benzodiazepines act on the brain and nerves to produce a calming effect. They enhance the effects of a certain natural chemical in the body (gamma-aminobutyric acid), a neurotransmitter chemical that nerves use to communicate with one another [34]. Diazepam is used to treat anxiety, insomnia, acute alcohol withdrawal and seizures. It is also used to relieve muscle spasms and to provide sedation before medical and dental procedures [35]. Diazepam is administered orally; but, it can also be given intravenously or intramuscularly. A short term use of this medication is generally safe and effective. However, the long term use of diazepam is very controversial, because of the potential of tolerance, dependence, withdrawal and other adverse effects [36]. Since diazepam is widely used in clinical and forensic cases, a rapid, simple and sensitive approach is necessary for its quantification in biological fluids and commercial pharmaceutical products [37].

**Scheme 1:**
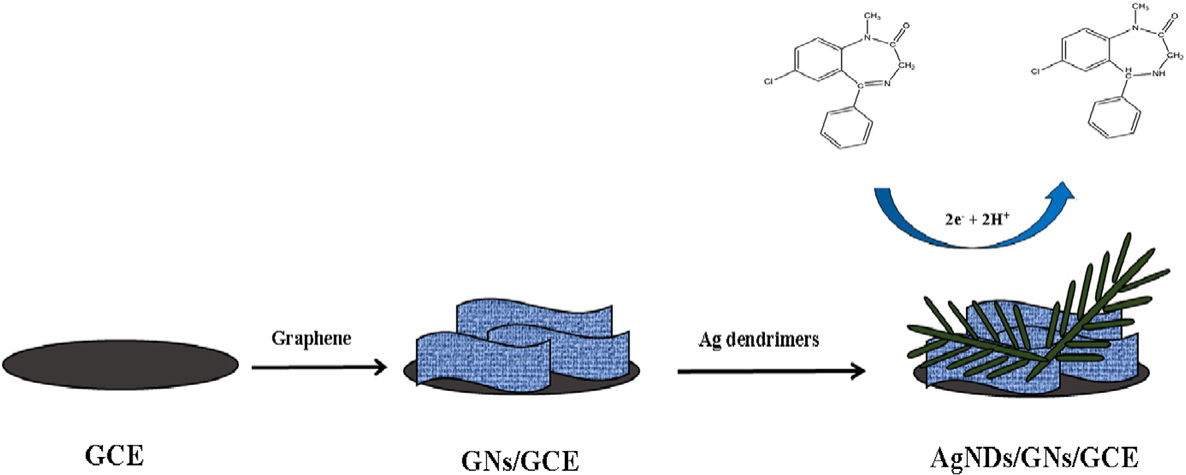
Schematic illustration for the preparation of AgNDs/GNs/GCE.

In the present paper, a facile and cost-effective approach was developed for determination of diazepam. This approach is based on Ag nanodendrimers supported by GNs modified glassy carbon electrode as illustrated in Scheme 1. This modified electrode benefit the advantages of graphene nanosheets and Ag nanodendrimers as good electrocatalysts, leading to the development of a high-performance electrochemical sensor for the analysis of diazepam and practical utility in clinical and quality control laboratories.

## 2 Experimental

### 2.1 Reagents and materials

Silver nitrate (AgNO_3_) was purchased from Merck (Germany). Diazepam, lorazepam, clonazepam and oxazepam were obtained from Zahravi Pharmaceutical Co. (Tabriz, Iran). Graphene nanosheets (Carbon content > 99.5 wt.%, diameter 1–20 μm, thickness 5–15 nm) were purchased from Xiamen Knano Graphit Technology Co. (China). Diazepam tablets (labeled amount 5 mg per tablet) and diazepam injection (10 mg/2 ml) were obtained from CHEMI DAROU Pharmaceutical Co. (Tehran, Iran) and Caspian Tamin Pharmaceutical Co. (Rasht, Iran), respectively. All other chemicals were of pure analytical grade and were used as received. Stock standard solutions of diazepam and other pharmaceutical were prepared by dissolving reagents in mixture of methanol–H_2_O (3:2 v/v). All these solutions were maintained at 4 °C in the dark. Aliquots from the stock solution were used for preparation of working solutions.

### 2.2 Instrumentation

Electrochemical measurements were carried out using the Potentiostat/Galvanostat Autolab PGSTAT30 (Eco Chemie B.V., The Netherlands) controlled by GPES 4.9 software. EIS experiments were performed using EG&G model parstat 263 Potentiostat/Galvanostat controlled by a PC. A classical three-electrode system was employed, comprising a modified glassy carbon electrode (GCE) with the diameter of 2 mm as working electrode, a platinum wire as auxiliary electrode and a saturated calomel electrode (SCE) as the reference electrode. All electrodes were purchased from Azar electrode Co. (Iran).

Cyclic voltammetry (CV) and differential pulse voltammetry (DPV) techniques were applied to study of the electrochemical behavior of diazepam. Differential pulse voltammetric experiments were carried out using a modulation time of 0.05 s, an interval time of 0.5 s, a modulation amplitude of 0.025 V and a scan rate of 0.01 V/s over a potential range of − 0.9 to − 1.2 V vs SCE.

Scanning electron microscopy images were obtained from a MIRA3 TESCAN made in Czech Republic. The XRD patterns of all electrode surfaces were recorded on a X-ray diffractometer (D500 S) using Cu Ka (k = 1.54 Å) radiation source (30–40 KV and 40–50 MA) in the range of 2θ = 35–85^0^. All electrochemical experiments were carried out at room temperature.

### 2.3 Preparation of real samples

The blood samples were taken from the healthy volunteer, 2 h after oral administration of the diazepam (the volunteer consumed no type of drugs which could interfere for benzodiazepines). In order to remove the proteins precipitate from blood plasma, 1 mL of plasma sample was mixed with 1 mL of methanol and vortexed for 60 s. Then, this solution was centrifuged for 5 min. The separated supernatant was collected in a 20 mL volumetric flask and diluted with phosphate buffer solution (pH 7).

To prepare the solutions of diazepam commercial samples, the tablet and injection samples were purchased from local drug stores. Ten tablets were weighed to determine their average mass and finely grounded into a powder. An accurately weighed portion of the resulting powder corresponding to the average mass of one tablet was transferred to a volumetric flask and dissolved in mixtures of methanol: H_2_O (3:2 v/v). This solution was stirred for 10 min by a magnetic stirrer and sonicated for 15 min. Then, the solution was filtered and diluted with phosphate buffer solution (pH 7) to an appropriate concentration. The injection samples were also prepared in the same way.

### 2.4 Preparation of AgNDs/GNs modified GCE

Prior to modification, the glassy carbon electrode was carefully cleaned by polishing with alumina slurry, subsequently washed ultrasonically in water for 5 min. After that, 3 μL of dimethylformamide (DMF) suspension containing 5 mg/ml of GNs was dropped on top of the GC electrode and dried in air to obtain the GNs/GCE.

AgNDs were electrochemically deposited on the surface of GNs/GCE using the potentiostatic method, without adding any surfactant and additional reagent, according to a reported procedure in literature [20]. The precursor solution was prepared by dissolving an amount of 10 μM AgNO_3_ in 0.1 M KNO_3_ solution. The deposition was performed in this solution by applying a potential of − 0.3 V to working electrode for 120 s. In order to remove the trapped nitrate anions in the structure of AgNDs, the modified electrodes were thoroughly rinsed several times with distilled water. Finally, the modified surface of the electrodes was dried at room temperature and used for later measurements.

## 3 Results and discussion

### 3.1 Morphological characterization of AgNDs/GNs/GCE

**Fig. 1:**
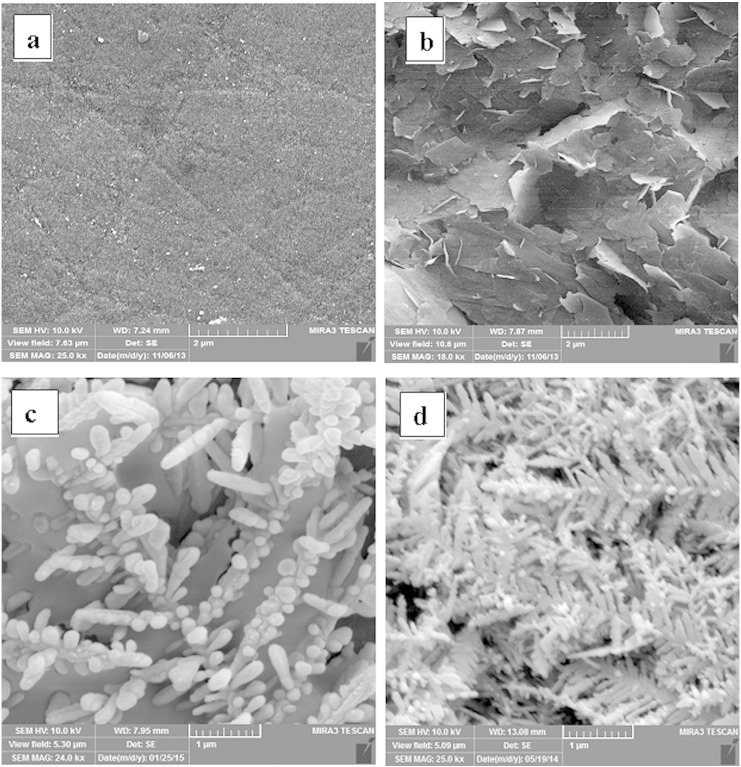
SEM images of (a) bare GCE (b) GNs/GCE (c) AgNDs/GNs/GCE synthesized under applied potential − 0.1 V and (d) − 0.3 V.

Characterization of nanostructures has an important role to describe their morphology, such as shape, size and spatial distribution. Thus, the morphology of modified electrodes was characterized using SEM technique and images shown in Fig. 1. As can be seen in Fig. 1a, the bare GC electrode has a uniform and smooth surface while graphene nanosheets modified electrode (Fig. 1b) has a wrinkled and crumpled surface. The rough structure of the GNs/GCE not only provides high specific surface area and high electron transfer rate but also gives more opportunities for further modification due to the large rough surface as scaffold.

The synthesis of the AgNDs under different applied potentials was investigated and SEM images of the top view of electrodes exhibited a nanodendritic structure (Fig. 1c–d). The AgNDs modified electrode has a uniform surface topography with cross-like and three-dimensional structure. Nanodendritic structure is consisting of a long central stem and sharp lateral branches, which branches are parallel to each other, forming a symmetric structure. At applied potential of − 0.1 V (Fig. 1c), the density and dimension of Ag nanodendrimers are low while, at the applied potential of − 0.3 V (Fig. 1d) Ag nanodendrimers are denser with larger dimensions. At potential of − 0.3 V, the length of trunks is around 4–5 μm and the length of the branches is about 0.7 μm. This dendritic structure could significantly increase the electrode effective surface area and improve its electrocatalytic activity.

**Fig. 2:**
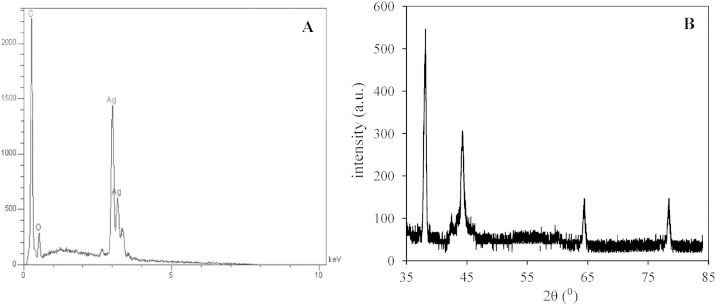
(A) EDX analysis and (B) X-ray diffraction pattern of AgNDs/GNs modified GCE.

Energy dispersive X-ray (EDX) spectroscopy was used to analyze the elemental composition of the AgNDs/GNs/GC electrode surface (Fig. 2A). The EDX spectrum displayed a very strong peak at 0.28 keV corresponding to C Kα due to the carbon atoms of the GNs. Also, two prevalent peaks were observed at 3.00 and 3.16 keV from Ag Lα_1_ and Ag Lβ_1_, confirming the presence of metallic Ag (from AgNDs) on the surface of graphene nanosheets. The amount of loaded Ag was about 82.8wt.% of total weight.

The successful synthesis of the Ag nanodendrimers was also confirmed by XRD analysis and the result was shown in Fig. 2B. In the XRD spectra, the four diffraction peaks at corresponding values of 38.16, 44.3, 64.43 and 78.4^0^ were observed that could be indexed to the (111), (200), (220) and (311) planes, respectively. The typical peaks of metallic Ag demonstrated that Ag has been decorated on the electrode surface in metallic state.

### 3.2 Characterization of AgNDs/GNs/GCE by electrochemical impedance spectroscopy (EIS) and cyclic voltammetry (CV)

**Fig. 3:**
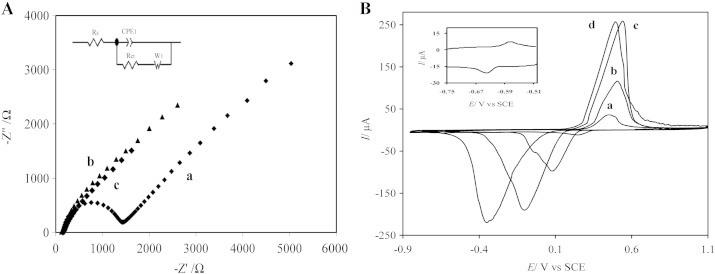
(A). EIS for (a) bare GCE, (b) GNs/GCE and (c) AgNDs/GNs/GCE in a solution of 10 mM [Fe(CN)_6_]^3 −/4 −^ and 0.1 M KCl with the frequencies swept from 2 × 10^6^ to 0.1 Hz under a constant DC potential of + 0.2 V condition. (B). Cyclic voltammograms of the AgNDs/GNs/GCE in 0.1 M PBS with various pH a) pH 3, b) pH 5, c) pH 7 and d) pH 9 at a scan rate of 50 mV/s.

EIS is a powerful and non-destructive method that can provide exact information about the interfacial properties of the electrode surface. The EIS measurements of bare and modified electrodes were performed using 10 mM [Fe(CN)_6_]^3 −/4 −^ redox couples as the electrochemical probe during the process. Fig. 3A presents the Nyquist plots of the bare GCE (a), GNs/GCE (b) and AgNDs/ GNs/GCE (c). The terms R_s_, CPE_1_, R_ct_ and W_1_ in the inset of Fig. 3A refer to solution resistance, double layer capacity, electron transfer resistance and specific electrochemical element of diffusion or Warburg element, respectively. The semicircle domain of impedance spectra, which can be observed at higher frequencies, corresponds to the electron transfer resistance. On the bare electrode (curve a), the charge transfer resistance, can be estimated to be 1405 Ω. On the GNs/GC electrode (curve b), this value dramatically decreased to 153 Ω, due to high conductivity of graphene nanosheets on the electrode surface. After electrodeposition of AgNDs on the GNs/GC electrode surface, the R_ct_ value was decreased to 104 Ω (curve c), due to the excellent conductivity and large surface area of AgNDs, which could increase the electron transfer rate between electrode surface and probe and decrease electron transfer resistance. These results demonstrated that immobilization of AgNDs/GNs on the surface of GC electrode highly reduces the electron transfer resistance.

The electrochemical behavior of the AgNDs/GNs modified GC electrode was investigated by CV to obtain straight and defined evidence about Ag nanodendrimers intrinsic properties. In order to this study, the cyclic voltammograms of the modified electrode were recorded in 0.1 M PBS solutions with various pH and results were presented in Fig. 3B.

Two set of anodic/cathodic peaks were appeared on the voltammograms, the first one was a high and sharp redox peak at positive potential and another one was a very broad and weak redox peak at more negative potential (inset in Fig. 3B). These redox peaks were attributed to adsorption/desorption of hydroxide ion on Ag that this transformation occurs according to the following reaction [38], [39]:

$$2{OH}_{\mathrm{ads}}^{-}+2Ag\to {Ag}_{2}O_{\mathrm{surf}}+H_{2}O+2e^{-}\text{.}$$

As can be seen, these peaks are pH dependent and shift negatively with increasing of pH and their peak current increase too. Also, the stability of the AgNDs/GNs/GC electrode was studied by repetitious voltammetric scanning at the scan rate of 50 mV/s in various pHs. Based on obtained results the AgNDs/GNs/GC electrode in natural and basic solutions is more stable than acidic solution and using of pH ≥ 7 for the electroanalysis application is reasonable.

### 3.3 Electrochemical behavior of diazepam on the modified electrodes

**Fig. 4:**
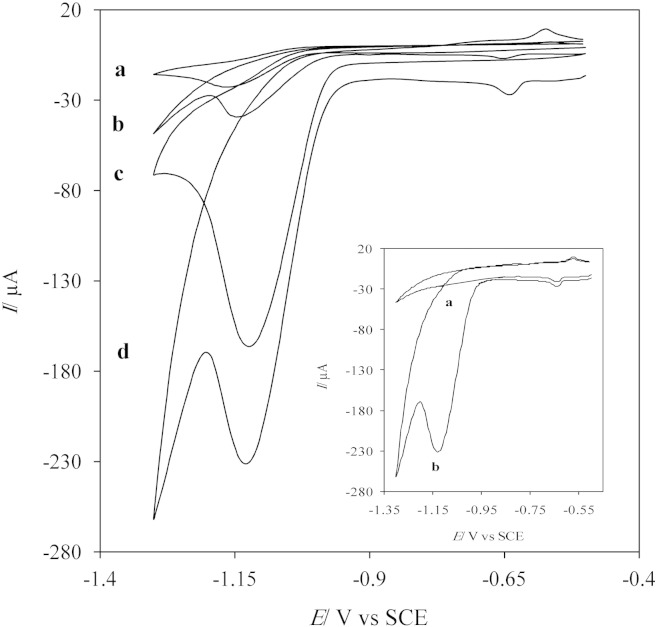
CVs of (a) bare GCE, (b) AgNDs/GCE, (c) GNs/GCE, (d) AgNDs/GNs/GCE in 0.1 M PBS pH 7 containing 3 mM DZP at a scan rate of 50 mV/s. Inset: CVs of the AgNDs/GNs/GCE in absence (a) and presence (b) of 3 mM DZP in 0.1 M PBS pH 7 at scan rate of 50 mV/s.

The electrochemical reduction of diazepam on bare GCE, AgNDs/GCE, GNs/GCE and AgNDs/GNs/GCE was investigated by cyclic voltammetry in 0.1 M PBS (pH 7) containing of 3 mM diazepam (Fig. 4).

From Fig. 4, it can be observed that: the broad and weak reduction peak of DZP appears at bare GCE (curve a) at potential of − 1.15 V. It indicates a poor electron transfer at the interface of this electrode. While, the AgNDs/GCE (curve b) displays a higher peak current and a positive potential shift for DZP reduction compared to bare GCE. The growth of AgNDs with very sharp edge sites on surface of the GCE improve the specific surface area and the active sites, leading to increase of the diazepam reduction current. Also, the peak current of DZP reduction on the GNs/GCE (curve c) increase significantly. This improved response could be related to high surface area, high conductivity and electrocatalytic activity of graphene nanosheets. Totally, compared to these three electrodes, the performance of AgNDs/GNs/GCE (curve d) is remarkable. The AgNDs/GNs/GC electrode extraordinary improves sensitivity of diazepam detection due to synergistic effects of graphene and Ag nanodendrimers.

**Scheme 2:**
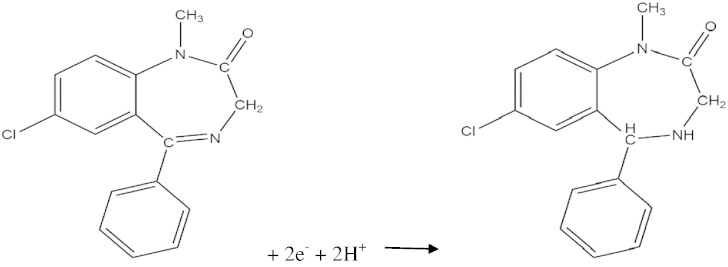
The electroreduction mechanism of diazepam.

In all electrodes, in the forward sweep, with potential scanning from − 0.4 to − 1.3, one reduction peak was seen at − 1.15 V and in the reverse sweep, no oxidation peak was observed. This reduction peak is due to reduction of azomethine functional group in the position four of the benzodiazepine ring according to the Scheme 2 [35], [40]:

It is clear that two electrons and two protons are necessary for reduction of the C=N group to the saturated group.

On the other hand, CVs of the AgNDs/GNs/GC electrode were recorded in absent and presence of 3 mM DZP (inset in Fig. 4). As can be observed, this electrode shows no peak in absent of DZP in the studied potential range, whereas due to high electroactivity, large specific surface area and high conductivity of AgNDs/GNs one sharp reduction peak was observed in presence of DZP.

### 3.4 Influence of solution pH

**Fig. 5:**
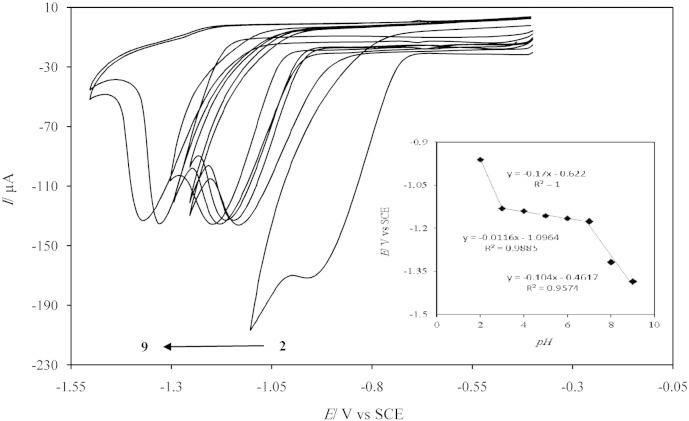
Cyclic voltammograms of the AgNDs/GNs/GCE in 0.1 M PBS with various pHs (from 2 to 9: 2, 3, 4, 5, 6, 7, 8 and 9) containing 1 mM DZP at scan rate of 50 mV/s. Inset: the plot of peak potential versus solution pH.

Based on Scheme 2, two protons and two electrons are postulated during DZP reduction; this obviously shows that the solution pH has a strong influence on the electrochemical reaction. So, the effect of solution pH on the peak current (I_p_) and peak potential (E_p_) of DZP reduction was investigated at various pH values from 2 to 9 and the results are shown in Fig. 5.

The results display that the peak potential is shifted toward more negative values with increase of pH. This behavior demonstrates that protons are consumed in the electrochemical reduction of DZP. Also, the results indicate that the plot of peak potential against pH has three linear zones with two breaks at 3.0 and 7.0 (inset in Fig. 5). The pK_a_ of this drug is found to be near 3.0 associated with N-4 protonation which is near the first break [41].

While, the peak currents of reduction are nearly equal in alkaline and neutral medium, but pH 2 shows a high peak current, due to a large number of protons in acidic medium. Although, a maximum peak current is achieved at pH 2, in order to analyze DZP in biological samples and also suitable stability of the modified electrode in pH ≥ 7, pH 7 is selected as suitable pH for latter experiments.

### 3.5 Influence of scan rate

**Fig. 6:**
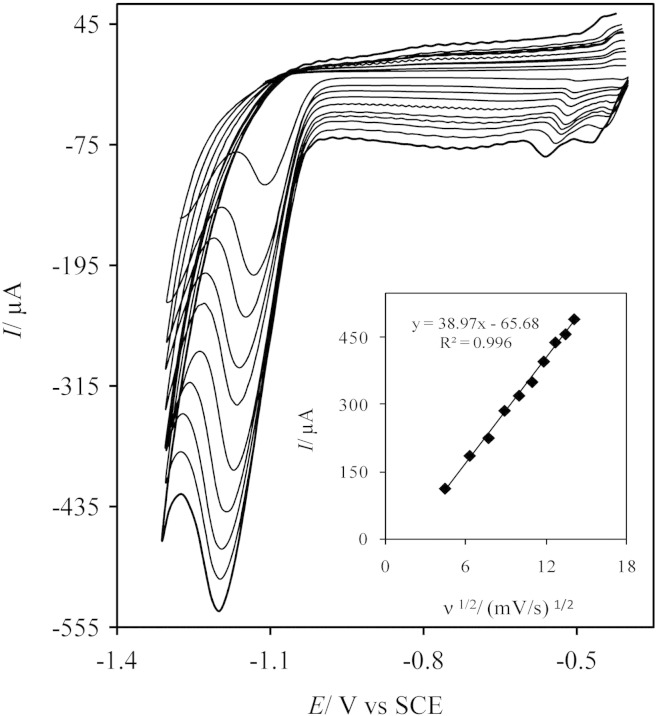
CVs of the AgNDs/GNs/GCE with different scan rates in 0.1 M PBS pH 7 containing 3 mM DZP. Inset: the plot of the reduction peak current versus the square root of scan rates. The scan rates are 20, 40, 60, 80, 100, 120, 140, 160, 180 and 200 mV/s from inside to outside, respectively.

The relationship between the current and potential of peak and scan rate gives important information about the electrochemical mechanism of the electrode process. Fig. 6 exhibits the obtained cyclic voltammograms on the AgNDs/GNs/GCE at various scan rates in 0.1 M PBS (pH 7) containing of 3 mM DZP. The recorded cyclic voltammograms show that reduction peak potentials shift to more negative amounts and the corresponding peak currents increase with increasing of scan rate. As seen in the inset of Fig. 6, the reduction peak currents is directly proportional to the square root of scan rates between 20 and 200 mVs^− 1^ with the correlation coefficients of 0.996. This behavior demonstrates that the reduction process is a diffusion-controlled one.

In addition, the electron transfer coefficient (α) can be obtained from the slope of Ep vs. Log ν plot. For an irreversible process, the cathodic peak potential can be represented by the Eq. (1) [42].

(1) $$E_{p}=\left(b/2\right)\log v+\mathrm{constant}$$

Here, b is Tafel slope (b = 2.303RT/αn_a_F, where n_a_ is the number of electrons involved in the rate-determining step and F is Faraday constant). On the basis of Eq. (1), the slope of Ep versus logν plot is b/2 which is obtained 162.8 mV/decade for DZP (the plot not shown). By assuming one-electron in rate-determining step the amount of (α) will be 0.36.

We also evaluated the diffusion coefficient (D) for DZP reduction process on the AgNDs/GNs/GCE. For a totally irreversible diffusion-controlled electrode process, the Randles–Sevcik equation can be used for evaluation of D [42]:

(2) $$I_{p}=2.69\times 10^{5}{ACn}^{3/2}D^{1/2}\nu^{1/2}$$

where n, A and C are the number of electrons involved in the thorough reduction process, surface area of the electrode and concentration of DZP, respectively. According to Eq. (2), the plot of I_p_ vs. ν^1/2^ should be linear that assuming n = 2, diffusion coefficient was obtained 3.06 × 10^− 6^ cm^2^/s.

The standard heterogeneous rate constant (k_s_) of diazepam on the AgNDs/GNs/GCE was calculated using the rotating disk electrode (RDE) voltammetry technique. The RDE experiments were performed at various rotation rates from 100 to 800 rpm and at scan rate of 20 mV/s. The recorded RDE voltammograms (Figure not shown) exhibit an increase in the current density with increase of the rotation rate, which is proportional to the square root of the rotation rate.

The RDE data were analyzed using the Koutecky–Levich plots (1/J vs 1/ω^1/2^) and the slope of plot was used to compute k_s_:

(3) $$\frac{1}{j}=\frac{1}{\mathrm{nFkC}}+\frac{1}{0.62\;\mathrm{nF}v^{-1/6}D^{2/3}C\omega^{1/2}}$$

where j is the measured current density, υ is the kinematic viscosity of the solution, ω is the rotation rate and other parameters are common parameters in literature. According of Eq. (3) and the slope of plot 1/J vs. 1/ ω^1/2^, the k_s_ was obtained 3.9 × 10^− 4^ cm/s.

### 3.6 Determination of diazepam

The charging current contribution on the background current is a limiting factor in analytical determination so, to overcome this problem, differential pulse voltammometry method was used for determination of diazepam. The reduction peak currents of DZP at various concentrations on the AgNDs/GNs/GCE were recorded in 0.1 M PBS (pH 7) solutions.

**Fig. 7:**
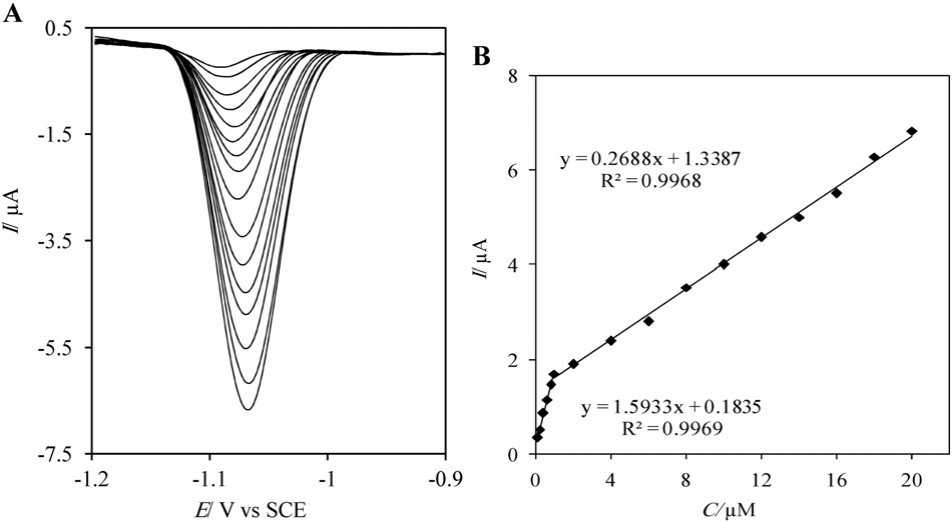
(A) DPVs of the AgNDs/GNs/GCE in 0.1 M PBS pH 7 with various concentrations of diazepam. (B) The calibration graphs derived from the DPVs (I/μA = 1.5933[DZP]/μM + 0.1835, R^2^ = 0.9969 and I/μA = 0.2688[DZP]/μM + 1.3387, R^2^ = 0.9968).

Fig. 7A displays that the height of the cathodic peak current increases with increasing of DZP concentration. The reduction peak current versus concentrations of diazepam was plotted (Fig. 7B), which the calibration curve showed two linear regions with different slopes. The first linear portion was from 0.1 to 1.0 μM and the second linear segment was up to 20.0 μM. The limit of detection (LOD) for differential pulse voltammetry method was calculated as 8.56 × 10^− 8^ M by using the first linear portion. The LOD was obtained by the equation: *y*_*LOD*_ = *y*_*B*_ + 3*S*_*y*/*x*_ and regression equation: current/μA = 0.1835 + 1.5933[DZP]/μM, R^2^ = 0.9969. While *y*_*B*_ is signal of the blank (here intercept of calibration curve), *S*_*y*/*x*_ standard deviation of blank (here standard deviation of the calibration curve) [43]. The results of the proposed sensor are comparable with other DZP sensors that were displayed in Table 1.

**Table 1:** Comparison of different modified electrodes for diazepam determination.

Electrode	Linear range	Detection limit	Methods	References
Lead film electrode	5 × 10^− 9^–5 × 10^− 7^ M	2.0 × 10^− 9^ M	Adsorptive stripping voltammetry	[34]
Modified carbon paste electrode	0.025–3.0 μg/mL	0.021 μg/mL	Differential pulse voltammetry	[36]
Unmodified SPCE^a^	7.1–285 μg/mL	1.8 μg/mL	Adsorptive stripping voltammetry	[35]
SngCE^b^ modified with BENT^c^	0.028–0.256 μg/mL	4.0 ng/mL	SWAdCSV^d^	[37]
DME^e^	5.6 × 10^− 8^–8.8 × 10^− 6^ and 8.8 × 10^− 6^–2.0 × 10^− 4^ M	9.6 × 10^− 9^ M	Polarography	[44]
AgNDs/GNs/GCE	1.0 × 10^− 7^–1.0 × 10^− 6^ and 1.0 × 10^− 6^–20 × 10^− 6^ M	8.56 × 10^− 8^ M	Differential pulse voltammetry	This work

a Screen-printed carbon electrode.

b Sonogel-Carbon electrode.

c Bentonite.

d Square wave adsorptive cathodic stripping voltammetry.

e Dropping mercury electrode.

### 3.7 Repeatability, stability and interference study

To investigative the repeatability of the proposed electrode, CVs of 3 mM diazepam in 0.1 M PBS (pH 7) were recorded for 6 replicate measurements under same condition. The relative standard deviation (RSD) for the reduction peak current and potential of diazepam was calculated and values of 6.57% and 5.1% were obtained, respectively. Thus, this modified electrode was found to exhibit excellent repeatability.

Another advantage of the AgNDs/GNs/GC electrode is its stability. The stability of modified electrode was examined using cyclic voltammetry in the 0.1 M PBS (pH 7) solution containing 3 mM DZP and the results were recorded for 2 weeks, every day. The plot of reduction peak current vs. time showed no significantly changes in the peak current during the studied time. The stability of electrode response demonstrated the long-term applicability of the AgNDs/GNs/GC electrode.

Furthermore, the interference of some drugs from benzodiazepines family such as oxazepam, lorazepam and clonazepam as potential interferents of DZP on the AgNDs/GNs/GCE was studied. A fixed amount of diazepam was mixed with these drugs. The results indicated these substances were interfering on diazepam determination, because these drugs have same structures and their reduction potentials are close to diazepam. They produce voltammetric peaks overlapping with the diazepam, influencing the peak potential and current response of DZP.

### 3.8 Real sample analysis

In order to evaluate the applicability and feasibility of the presented electrode, this electrode was used for determination of DZP in real samples (tablet, injection and plasma samples). The standard addition method was used for the analysis of tablet and injection samples, with three replicate measurements for each sample. From plot of the peak currents versus DZP concentration, contents of DZP in samples were obtained. Then, the t-test was used for the statistical comparison of the obtained results by proposed method and certificated value. The t value at a confidence level of 95% is 4.3 (α = 0.05) and the obtained t values were smaller than the critical one, it revealed that the obtained contents of diazepam by present method are in good agreement with the claimed value. The obtained results given in Table 2.

**Table 2:** Results of determination of diazepam in tablet and injection samples.

Sample	Amount labeled	Amount found a	RSD%	t^b^
Tablet	5 mg per tablet	4.75	1.05	1.9
Injection	10 mg/2 ml	9.65	0.51	2.7

a Average of three replicate determination.

b t Critical = 4.3 at a confidence level of 95%.

The plasma samples were spiked with known amounts of diazepam. The differential pulse voltammograms were recorded under exactly identical conditions as were employed for the preparation of calibration curve. According to the presented results in Table 3, good recoveries for diazepam were obtained in samples, indicating the suitability of the proposed methodology for direct analysis of diazepam in blood plasma samples.

**Table 3:** Results of the recovery analysis of DZP in human blood plasma samples.

Sample	Amount added (μM)	Amount found (μM)	Recovery (%)
Blood plasma (1)	–	Not detected	–
	2.0	2.1	105
	4.0	3.9	97.5
Blood plasma (2)	–	Not detected	–
	2.0	2.2	110
	4.0	4.3	107.5

## 4 Conclusion

In this research an effective method has been introduced for fabricating a diazepam sensor. This sensor utilize the synergetic influence of Ag nanodendrimers and graphene nanosheets. It was shown that the AgNDs can be synthesized on the GNs/GCE without any additional reducing reagent. The AgNDs/GNs possesses high electrocatalytic activity toward diazepam reduction and improve analytical performance of sensor significantly. It is due to exclusive properties of Ag nanodendrimers and graphene nanosheets such as high amounts of sharp edges as electroactive sites along with large efficient surface area that enhance the electron-transfer rate of the reduction processes. A good linear range and an excellent detection limit are obtained using this modified electrode. Additionally, determination of DZP in tablet and injection samples is successfully performed by the standard addition method and in human blood plasma with high recovery.

This sensor shows high repeatability and stability and offers several advantages such as simplicity, rapidity and low cost that can be considered as merits of the proposed sensor in the determination of the analytes.
